# Exploring the association between ceramide, phosphatidylcholine, and COPD prevalence and incidence: a FINRISK population-based cohort study

**DOI:** 10.1186/s12890-025-03884-7

**Published:** 2025-10-15

**Authors:** Mohammadreza Shoghli, Juha Sinisalo, A. Inkeri Lokki, Mitja Lääperi, Marja-Liisa Lokki, Mika Hilvo, Antti Jylhä, Jaakko Tuomilehto, Reijo Laaksonen

**Affiliations:** 1 Department of Population Health, University of Helsinki, Helsinki, 00014 Finland; 2 Department of Bacteriology and Immunology, University of Helsinki, Helsinki, 00014 Finland; 3 Heart and Lung Center, Helsinki University Hospital, University of Helsinki, Helsinki, 00014 Finland; 4 Zora Biosciences Oy, Espoo, Espoo, 02620 Finland; 5 Department of Pathology, University of Helsinki, Helsinki, 00290 Finland; 6 VTT Technical Research Centre of Finland, Espoo, 02044 Finland; 7 Population Health Unit, Finnish Institute for Health and Welfare, Helsinki, 00271 Finland; 8 Department of Public Health, University of Helsinki, Helsinki, 00014 Finland; 9 Department of International Health, National School of Public Health, Instituto de Salud Carlos III, Madrid, 28029 Spain; 10 Finnish Cardiovascular Research Centre, University of Tampere, Tampere, 33521 Finland

**Keywords:** COPD, Ceramides, Phosphatidylcholine, Lipid biomarkers, CERT scores, Prevalence, Incidence, FINRISK cohort, Smoking

## Abstract

**Background:**

Ceramides (Cers) and phosphatidylcholines (PCs) are potential lipid biomarkers in obstructive pulmonary disease (COPD). Even though they are linked to inflammation and lipid dysregulation, little is known about how these factors affect the prevalence and incidence of COPD in population-based cohorts. This study investigates these associations, addressing knowledge gaps regarding the interplay of Cers, PCs, and COPD risk, focusing on sex-specific differences and smoking.

**Methods:**

This observational study analysed data from the population-based FINRISK 2002 cohort, with 7,722 participants for prevalence and 7,662 for incidence analyses. Logistic regression models were used to assess associations between lipid biomarkers and prevalent COPD, while Cox regression models were applied for incident COPD. CERT1 and CERT2 (Cardiovascular Event Risk Test 1 and 2) are lipid-based scores derived from ceramide (Cer) ratios that estimate cardiovascular risk; in this study, they were used to examine their association with COPD. Kaplan-Meier curves were used to evaluate the impact of CERT scores on COPD risk, stratified by smoking status.

**Results:**

Elevated CERT1 and CERT2 scores were associated with both prevalent and incident COPD. For CERT1, the association with prevalent COPD was significant (univariable OR = 1.81, 95% CI: 1.41–2.33, *p *= < 0.001), as was the association with incident COPD (univariable HR = 1.33, 95% CI: 1.16–1.53, *p* = < 0.001). CERT2 was also significantly associated with prevalent COPD (adjusted OR = 1.57, 95% CI: 1.15–2.16, *p* = 0.005) and with incident COPD (univariable HR = 1.53, 95% CI: 1.32–1.77, *p* = < 0.001). PC species (14:0/22:6) was significantly associated with a lower risk of incident COPD (adjusted HR = 0.85, 95% CI: 0.73–0.98, *p* = 0.023). The Cer(d18:1/18:0)/PC (14:0/22:6) ratio was associated with both prevalent COPD (adjusted OR = 1.37, 95% CI: 1.01–1.86, *p* = 0.041) and incident COPD (HR = 1.24, 95% CI: 1.07–1.44, *p* = 0.004). Smokers had an elevated risk of COPD with increasing CERT scores.

**Conclusion:**

These findings support the role of lipid biomarkers, particularly Cers and CERT scores, in improving COPD risk prediction and management, with potential implications for targeted interventions in smokers.

**Supplementary Information:**

The online version contains supplementary material available at 10.1186/s12890-025-03884-7.

## Introduction

Chronic Obstructive Pulmonary Disease (COPD) is a significant global health burden, contributing to both morbidity and mortality worldwide. Chronic respiratory symptoms and reduced airflow result from prolonged exposure to harmful particles or gases, leading to abnormalities in the airways and alveoli [1]. Other contributing risk factors include genetic predisposition, sex differences, socioeconomic status, early-life disadvantages, biomass fuel smoke, occupational hazards, and air pollution, underscoring the need for improved diagnostic and preventive strategies [2]. In 2019, COPD accounted for 212.3 million prevalent cases, 3.3 million deaths, and 74.4 million Disability-Adjusted Life Years (DALYs) [3]. The systemic complications of COPD, such as pulmonary hypertension and muscle dysfunction, are driven by chronic inflammation and physical inactivity, further highlighting the importance of advancing diagnostic tools and therapeutic strategies [1, 3].

Recent studies have emphasized the significant role of lipid metabolism in the pathogenesis of COPD, particularly in regulating immune responses and inflammation. Ceramides (Cers), key lipid components of cell membranes, are now recognized for their involvement in maintaining lung function and contributing to the inflammatory processes characteristic of COPD [4]. Disruption of lipid raft structure and function, particularly in smokers, contributes to COPD pathogenesis by promoting bacterial colonization in the bronchi and increasing inflammation [4]. Phospholipids, including sphingomyelins and glycerophospholipids, serve as essential components of cell membranes [5]. Recent studies in humans and mice exposed to cigarette smoke have demonstrated that it activates sphingomyelinases, including acid sphingomyelinase (aSMase) and neutral sphingomyelinase 2 (nSMase2), resulting in elevated ceramide levels in both lung tissue and the bloodstream [6, 7]. The accumulation of ceramides contributes to oxidative stress, chronic inflammation, and apoptosis of airway epithelial cells, as well as the release of ceramide-rich microparticles and extracellular vesicles from pulmonary macrophages that impair endothelial function—processes implicated in the development and progression of COPD [6, 7].

Cers are crucial bioactive lipids that play a role in multiple cellular functions, including apoptosis, necrosis, and autophagy-related cell death [8]. Elevated Cer levels, particularly Cer (d18:1/16:0), are observed in response to cigarette smoke exposure, which may lead to mitochondrial damage and PINK1-mediated necroptosis [9]. Increased Cer levels lead to apoptosis in pulmonary epithelial cells, contributing to alveolar destruction—a hallmark of emphysema in COPD [8].

The CERT1 and CERT2 (Cardiovascular Event Risk Tests 1 & 2) have been significantly associated with inflammatory markers such as high-sensitivity C-reactive protein (hs-CRP) and interleukin-6 (IL-6) and have been linked to various cardiovascular disease (CVD) outcomes, highlighting their relevance as inflammation-related biomarkers [10]. CERT1 and CERT2 are valuable tools for both primary and secondary prevention of CVD, including coronary artery disease, chronic heart failure, and stroke [11]. Despite growing awareness of the connection between lipid metabolism and COPD, few large-scale population-based cohort studies have assessed the contribution of ceramides and phosphatidylcholines (PCs) to COPD incidence and prevalence. This study aimed to evaluate the association between specific Cer and PC species and the prevalence and incidence of COPD in a large-scale population cohort.

## Materials and methods

### Objectives

The objective of this study was to investigate the association of specific Cer and PC species with the prevalence and incidence of COPD, with a focus on sex-specific differences and the interplay with smoking, using data from the population-based FINRISK 2002 cohort.

### Population characteristics

The FINRISK cohort comprises nationally representative, population-based health examination surveys conducted every five years in Finland since 1972. These surveys monitor risk factors for noncommunicable diseases (NCDs), including cardiovascular disease, diabetes, obesity, respiratory disease, and cancer. As a key component of Finland’s public health surveillance, the FINRISK cohorts provide valuable longitudinal data for epidemiological research, including studies on COPD in the Finnish population. By 2012, FINRISK had enrolled over 101,000 adults across six regions of Finland, with participation rates ranging from 57% to 94% [12].

The surveys support prevention programs, risk estimation, and national health policies. The study collected self-reported and measured data from the health exams, including biological samples, which were linked annually to national health registers. A validated method using liquid chromatography–mass spectrometry (LC-MS/MS) was applied to analyse distinct Cer and phospholipid species [13]. This comprehensive follow-up has enabled detailed longitudinal research on health status, behaviours, and disease occurrence. For the present prospective study on COPD in the FINRISK 2002 cohort, the median follow-up period was 15.8 years (IQR, 15.8–15.9 years).

Education was defined as low if the highest attained level was elementary school, basic education, lower secondary education, vocational school, or an equivalent qualification, and as high if it was upper secondary education or high school, non-university lower education, non-university higher education, or university education.

Two study designs were applied to investigate COPD:

1. Prevalence study: Included 7,722 participants, comprising 7,662 without COPD and 60 with prevalent COPD at baseline.
2. Incidence study: Included 7,662 participants free of COPD at baseline, of whom 7,457 remained unaffected, while 205 developed COPD during follow-up.

### Definition of prevalence and incidence of COPD and diagnosis criteria

The prevalence of COPD refers to the total number of diagnosed cases in the population at the time of the baseline assessment. The incidence of COPD refers to the new cases identified during the follow-up among individuals in the population who were not diagnosed with COPD at baseline. The COPD cases were identified using record linkage with the National Hospital Discharge Register or Death Register (incident cases only) and the national personal identification number.

The diagnosis of COPD in this study follows the Finnish national guidelines issued by the Finnish Medical Society Duodecim [14]. According to these guidelines, diagnosis is based on:

- Exposure to hazards (e.g., tobacco smoke, occupational exposures).
- Presence of respiratory symptoms (e.g., dyspnea, chronic cough, sputum production).
- Spirometry showing a post-bronchodilator FEV₁/FVC ratio < 0.70, indicating persistent airflow limitation 

In accordance with the GOLD 2023 recommendations [15], additional diagnostic considerations may include quantified smoking history (e.g., pack-years), occupational exposures, and a detailed assessment of respiratory symptoms. Although not specified in the Finnish guideline, a chest X-ray may be used to rule out other conditions. In individuals with overlapping features of asthma and COPD, the presence of asthma–COPD overlap (ACO) is considered during treatment planning.

### Laboratory measurements

Venous blood was drawn and centrifuged on-site, and the resulting serum was immediately frozen. Samples were then transported weekly on dry ice to the laboratory of the Finnish Institute for Health and Welfare (THL) for analysis. Blood collection followed a standardized protocol requiring a minimum fasting period of four hours, with sampling typically occurring between 11:00 and 19:00. This protocol was designed to minimize variability and is consistent with procedures established in previous FINRISK cohort studies, as documented in prior publications. A validated targeted liquid chromatography–tandem mass spectrometry (LC-MS/MS) method was used to quantify four ceramides—Cer (d18:1/16:0), Cer (d18:1/18:0), Cer (d18:1/24:0), and Cer (d18:1/24:1)—and three phosphatidylcholines—PC (14:0/22:6), PC (16:0/22:5), and PC (16:0/16:0). These analyses were performed using established protocols from Zora Biosciences Oy [16].

### Cardiovascular event risk test 1 (CERT1)

CERT1 is a measure developed to evaluate Cers and their relative ratios by comparing the concentrations of Cer (d18:1/16:0), Cer (d18:1/18:0), and Cer (d18:1/24:1) to that of Cer (d18:1/24:0). This approach provides insights into specific Cer components and their relative proportions [17]. Participants with concentrations or ratios in the fourth quartile were awarded two points, while those in the third quartile received one point. Individuals in the first and second quartiles were assigned zero points. The CERT1 score ranges from 0 to 12 [11]. For further details on how these metrics correlate with coronary heart disease risk, see Supplementary Fig. 1.

### Cardiovascular event risk test 2 (CERT2)

To improve upon CERT1 and incorporate the predictive potential of PCs for heart disease [16, 18], a refined version called CERT2 was developed. CERT2 includes one Cer ratio (Cer (d18:1/24:1)/(d18:1/24:0)), two Cer/PC ratios (Cer (d18:1/16:0)/PC (16:0/22:5) and Cer (d18:1/18:0)/PC (14:0/22:6)), and one standalone PC (PC 16:0/16:0). The scoring of CERT2 ranges from 0 to 12 points, based on the total points accumulated by the individual as depicted in Supplementary Fig. 1 .

### Quartile categorization of CERT1 and CERT2

To evaluate the associations of individual lipid components of CERT1 and CERT2 with COPD risk, each component lipid ratio was divided into quartiles based on its distribution in the FINRISK 2002 cohort (*N* = 7,722). The 25th, 50th (median), and 75th percentile values were applied to define the quartile boundaries. Each quartile comprises approximately 25% of the study population (*n* ≈ 1,930). These quartile-based categories were applied in both descriptive and survival (Kaplan–Meier) analyses to stratify COPD risk. Full percentile distributions for all individual Cer and PC ratios included in the CERT1 and CERT2 scores are presented in Supplementary Table 1.

The CERT1 and CERT2 scores were analyzed using their predefined risk categories: low, moderate, increased, and high risk, as described in previous studies [11]. 

### Statistical analysis

We described the study population using medians with interquartile ranges (IQRs) and counts and percentages. We compared the lipid concentrations and other variables between individuals affected with COPD and those unaffected using Mann-Whitney U tests and chi-squared tests. We modelled prevalent COPD using logistic regression models, while incident COPD was modelled using Cox regression, with age as the time scale. In both Table 2 and Table 3, estimates for CERT scores and Cer-related components were calculated per log-transformed standard deviation (SD), except for CERT1 and CERT2, which are shown per SD units.

To minimise the risk of overfitting, we followed the commonly accepted guideline of allowing 10 to 20 effective outcome events per covariate in each model.

#### Adjustments (Supplementary Table 2)

The prevalence models were adjusted for age and sex, or for age, current smoking, ex-smoking, hs-CRP, and prevalent asthma. The incidence models were stratified by sex and included adjustments for body mass index (BMI), current smoking, ex-smoking, education level (low vs. high), hs-CRP, and prevalent asthma. A model where current smoking was included as a stratifying variable, rather than a covariate, was also included.

#### Handling missing data

A small number of observations were excluded due to minor technical issues in lipid quantification. Specifically, three observations were excluded from the CERT1 analysis due to failed Cer measurements, and one additional observation was excluded from the CERT2 analysis due to a failed PC measurement. These exclusions, which are common in large-scale lipidomic studies, did not materially affect the analysis outcomes.

#### Kaplan-Meier visualization

Kaplan-Meier plots were generated to assess the association between CERT scores and the probability of incident COPD in both smokers and non-smokers. Survival probabilities over time were compared between groups based on CERT grades. Differences between survival curves were tested using the log-rank test.

A *p*-value below 0.05 was considered statistically significant. All analyses were performed using R software version 4.4.1 [19], with the ‘survival’ package for fitting Cox regression models [20] and the ‘survminer’ package for generating Kaplan-Meier plots [21].

## Results

### Baseline characteristics

Table 1 presents the baseline characteristics of participants with and without prevalent and incident COPD. Participants in both COPD groups were older than apparently healthy controls, with median ages of 64.3 years for prevalent and 58.0 years for incident COPD, compared with 48.5 and 48.0 years, respectively, in controls. Men predominated in both COPD groups, accounting for 72.7% of participants with incident COPD and 63.3% with prevalent COPD. Nearly half of the participants with prevalent COPD were ex-smokers. A high percentage of participants with incident COPD were current smokers (69.1% of men and 83.9% of women) at baseline. Asthma was more frequently observed among individuals who either had COPD at baseline or developed it during follow-up. Both prevalent and incident COPD were more common in individuals with lower educational attainment. Blood pressure was elevated in both groups. Participants with prevalent COPD had higher systolic blood pressure (145 mmHg) compared with those without COPD (131 mmHg), and a similar difference was observed in the incident COPD group (140 mmHg vs. 131 mmHg). Both COPD groups also had higher levels of serum hs-CRP and triglycerides than those without COPD. In addition, the concentration of apolipoprotein B (ApoB) elevated in people with incident COPD. The concentration of HDL cholesterol was slightly lower in men with prevalent COPD. Waist circumference was larger in both COPD groups, with a more pronounced increase observed in those with prevalent COPD. Average CERT1 and CERT2 scores were significantly higher in people with both prevalent and incident COPD than in those without COPD.

**Table 1:** Baseline characteristics of participants in prevalent (A) and incident (B) COPD study populations

(A) study on prevalence of COPD			
Variable	People without COPD (*N* = 7662)	People with COPD (*N* = 60)	*P*- value
Age (years)	48.5 (37.2–58.2)	64.3 (58.3–68.2)	<0.001
Men	3581 (46.7%)	38 (63.3%)	0.015
Higher education (%)	3300 (43.1%)	14 (23.3%)	0.003
Serum total cholesterol (mmol/L)	5.47 (4.84–6.21)	5.71 (4.94–6.23)	0.515
Serum HDL cholesterol (mmol/L) ^b^ Men	1.29 (1.09–1.54)	1.22 (1.04–1.43)	0.316
Serum HDL cholesterol (mmol/L) ^b^Women	1.59 (1.35–1.88)	1.66 (1.21–1.79)	0.355
Serum LDL cholesterol (mmol/L) ^c^	3.29 (2.71–3.90)	3.31 (2.61–3.70)	0.776
Serum triglycerides (mmol/L)	1.18 (0.85–1.69)	1.57 (1.14–2.23)	<0.001
ApoB (g/L) ^e^	0.96 (0.82–1.13)	0.97 (0.88–1.16)	0.161
BMI (Kg/m²) ^a^	26.3 (23.6–29.4)	28.4 (24.9–33.7)	0.002
Systolic blood pressure (mmHg)	131 (120–145)	145 (129–158)	0.388
Diastolic blood pressure (mmHg)	79 (71–86)	80 (73–90)	<0.001
Serum hs-CRP (mg/L) ^d^	1.13 (0.53–2.55)	2.32 (0.91–6.12)	<0.001
Waist circumference (cm) – Men	94.5 (87.0–102.5)	102.0 (94.9–113.0)	<0.001
Waist circumference (cm) – Women	82.0 (74.5–91.0)	97.5 (80.2–104.8)	<0.001
Current smoking – Men	1101 (30.7%)	13 (34.2%)	0.777
Current smoking – Women	851 (20.9%)	7 (31.8%)	0.318
Ex-Smoking – Men	995 (27.8%)	20 (52.6%)	<0.001
Ex-Smoking – Women	674 (16.5%)	7 (31.8%)	0.102
History of diabetes	436 (5.7%)	13 (21.7%)	<0.001
History of lipid – lowering drug treatment	562 (7.3%)	13 (21.7%)	<0.001
History of blood pressure-lowering drug treatment	1096 (14.3%)	27 (45.0%)	<0.001
History of asthma	619 (8.1%)	44 (73.3%)	<0.001
CERT1^f^	4 (2–7)	6 (5–9)	<0.001
CERT2^g^	6 (4–8)	8 (6–9)	<0.001
(B) Study on incidence of COPD			
Variable	People without COPD (*N*=7457)	People with COPD (*N*=205)	*P*-value
Age (years)	48.0 (37.0–57.9)	58.0 (51.8–64.3)	<0.001
Men	3432 (46.0 %)	149 (72.7 %)	<0.001
Higher education (%)	3265 (43.8 %)	35 (17.1 %)	<0.001
Serum total cholesterol (mmol/L)	5.47 (4.84–6.21)	5.72 (5.03–6.30)	0.019
Serum HDL cholesterol (mmol/L)^b^Men	1.29 (1.09–1.54)	1.28 (1.08–1.54)	0.877
Serum HDL cholesterol (mmol/L) ^b^ Women	1.59 (1.35–1.88)	1.41 (1.15–1.71)	0.002
Serum LDL cholesterol (mmol/L) ^c^	3.29 (2.70–3.89)	3.39 (2.96–4.17)	0.004
Serum triglycerides (mmol/L)	1.17 (0.85–1.68)	1.38 (1.01–1.92)	<0.001
ApoB (g/L) ^e^	0.96 (0.81–1.13)	1.02 (0.91–1.21)	<0.001
BMI (Kg/m²) ^a^	26.3 (23.6–29.4)	27.0 (23.4–29.3)	0.675
Systolic blood pressure (mmHg)	131 (120–145)	140 (123–155)	<0.001
Diastolic blood pressure (mmHg)	79 (71–86)	81 (74–89)	0.005
Serum hs-CRP (mg/L) ^d^	1.12 (0.52–2.49)	2.00 (0.94–4.09)	<0.001
Waist circumference (cm) – Men	94.5 (87.0–102.0)	97.2 (87.4–107.0)	0.053
Waist circumference (cm) – Women	82.0 (74.5–91.0)	86.2 (75.9–95.0)	0.103
Current smoking – Men	998 (29.1 %)	103 (69.1 %)	<0.001
Current smoking – Women	804 (20.0 %)	47 (83.9 %)	<0.001
Ex-Smoking – Men	956 (27.9 %)	39 (26.2 %)	0.723
Ex-Smoking – Women	669 (16.6 %)	5 (8.9 %)	0.174
History of diabetes	416 (5.6 %)	20 (9.8 %)	0.017
History of lipid – lowering drug treatment	534 (7.2 %)	28 (13.7 %)	<0.001
History of blood pressure-lowering drug treatment	1050 (14.1 %)	46 (22.4 %)	<0.001
History of asthma	585 (7.8 %)	34 (16.6 %)	<0.001
CERT1^f^	4 (2–7)	6 (3–9)	<0.001
CERT2^g^	6 (4–8)	7 (6–9)	<0.001

^a^*BMI* body mass index

^b^*HDL* high-density lipoprotein

^c^*LDL* low-density lipoprotein

^d^ *hs-CRP* high-sensitivity C-reactive protein

^e^ *ApoB* Apolipoprotein

^f^*CERT1* Cardiovascular Event Risk Test 1

^g ^*CERT2* Cardiovascular Event Risk Test 2

Values are presented as medians (IQRs) for continuous variables and counts (percentage in group) for categorical variables. Continuous variables were compared using Mann–Whitney U tests and categorical variables using chi-squared tests

### Prevalent COPD in participants aged ≥ 50 years (N = 59)

Because individuals with prevalent COPD were predominantly older (only one participant with COPD was under 50 years old), we restricted the analyses to participants aged 50 years or older. As shown in Supplementary Table 3, participants with prevalent COPD were predominantly male and had higher hs-CRP levels, larger waist circumference, elevated serum triglycerides, and higher CERT1 and CERT2 scores compared with those without COPD. Additionally, participants with COPD had a higher prevalence of diabetes, were more likely to use blood pressure–lowering medication, and more often had a history of asthma.

### Incident COPD in participants aged ≥ 50 years (N = 161)

Incident COPD was more common among older participants. Among those aged ≥ 50 with incident COPD (Supplementary Table 4 A), the median age was 60.7 years, and the majority were men. Compared with participants without COPD, those with incident COPD had significantly higher levels of serum triglycerides and hs-CRP. At baseline, a larger proportion of participants with incident COPD were current smokers, had a history of asthma, and had a lower educational attainment. Both CERT1 and CERT2 scores were also notably higher in this group.

### Incident COPD in participants aged <50 years (N = 44)

 Among participants aged < 50 years, 44 individuals (0.1%) developed incident COPD (see Supplementary Table 4B). Those who developed COPD were older (median age 43.3 years), and compared with those without incident COPD, had higher levels of serum triglycerides and hs-CRP and lower HDL cholesterol levels in women. Smoking and lower educational attainment at baseline were significantly more common among participants with COPD, and their CERT2 scores were also higher.

### Regression analysis of CERT scores and their components

Table 2 presents results from logistic and Cox regression analyses examining the association of CERT scores, Cer, and PC components for the prevalence of COPD (A) and the incidence (B). For the analysis of COPD prevalence, models were adjusted for age, hs-CRP, current smoking, former smoking, and prevalent asthma. For COPD incidence, Cox models were performed using age as the time scale, with stratification by sex, and adjustments for BMI, current smoking, former smoking, level of educational attainment, hs-CRP, and prevalent asthma (Model 1). An alternative model (Model 2) was also applied, in which smoking status was treated as a stratifying variable rather than a covariate, meaning that analyses were performed separately for smokers and non-smokers rather than adjusting for smoking as a covariate.

**Table 2:** Logistic and Cox regression results for CERT scores and components in prevalent (A) and incident (B) COPD

A. Study on prevalence of COPD (*N*=7722)						
Variable	Unadjusted OR (95%CI)	Unadjusted *P*-value	Adjusted OR (95%CI)	Adjusted *P*-value		
CERT1	1.81 (1.41 - 2.33)	<0.001	1.27 (0.96 - 1.70)	0.095		
CERT2	2.18 (1.67 - 2.87)	<0.001	1.57 (1.15 - 2.16)	0.005		
Cer (d18:1/16:0)	1.59 (1.25 -2.01)	<0.001	1.22 (0.92 - 1.62)	0.164		
Cer (d18:1/18:0)	1.72 (1.33 - 2.24)	<0.001	1.14 (0.85 - 1.54)	0.390		
Cer (d18:1/24:0)	1.41 (1.09 - 1.83)	0.009	1.17 (0.87 - 1.57)	0.304		
Cer (d18:1/24:1)	1.84 (1.44 - 2.34)	<0.001	1.39 (1.02 - 1.89)	0.036		
PC (14:0/22:6)	0.98 (0.76 - 1.26)	0.849	0.79 (0.60 - 1.05)	0.104		
PC (16:0/16:0)	1.52 (1.21 -1.89)	<0.001	1.15 (0.86 - 1.51)	0.343		
PC (16:0/22:5)	1.04 (0.81 -1.34)	0.765	1.00 (0.77 - 1.31)	0.990		
Cer (d18 :1/16:0)/Cer (d18:1/24:0) ratio	1.11 (0.86 -1.42)	0.415	1.04 (0.79 - 1.37)	0.779		
Cer (d18 :1/18:0)/Cer (d18:1/24:0) ratio	1.42 (1.10 -1.83)	0.008	1.02 (0.76 - 1.37)	0.921		
Cer (d18 :1/24:1)/Cer (d18:1/24:0) ratio	1.53 (1.18 -1.97)	0.001	1.20 (0.91 - 1.60)	0.192		
Cer (d18:1/16:0) /PC (16:0/22:5) ratio	1.52 (1.18 -1.95)	0.001	1.21 (0.91 - 1.62)	0.183		
Cer (d18:1/18:0) /PC (14:0/22:6) ratio	1.45 (1.13 -1.85)	0.003	1.37 (1.01 - 1.86)	0.041		
Cer (d18:1/18:0)/Cer (d18:1/16:0) ratio	1.39 (1.07–1.80)	0.014	0.98 (0.73–1.32)	0.905		
Model adjusted for age (log), current smoking, ex smoking, hs-CRP, prevalent asthma						
B. Study on incidence of COPD (*N*=7662)						
Variable	Unadjusted HR (95%CI)	Unadjusted *P*-value	Model 1 HR (95%CI)	Model 1 *P*-Value	Model 2 HR (95%CI)	Model 2 *P*-Value
CERT1	1.33 (1.16 - 1.53)	<0.001	1.15 (0.99 - 1.33)	0.060	1.14 (0.99 - 1.32)	0.067
CERT2	1.53 (1.32 - 1.77)	<0.001	1.14 (0.98 - 1.33)	0.085	1.14 (0.98 - 1.33)	0.081
Cer (d18:1/16:0)	1.30 (1.13 - 1.50)	<0.001	1.03 (0.89 - 1.19)	0.673	1.03 (0.89 - 1.19)	0.740
Cer (d18:1/18:0)	1.39 (1.20 - 1.61)	<0.001	1.14 (0.97 - 1.34)	0.115	1.13 (0.96 - 1.33)	0.137
Cer (d18:1/24:0)	1.22 (1.05 - 1.41)	0.008	0.96 (0.83 - 1.12)	0.615	0.95 (0.82 - 1.11)	0.536
Cer (d18:1/24:1)	1.38 (1.19 - 1.60)	<0.001	1.04 (0.89 - 1.21)	0.650	1.03 (0.88 - 1.21)	0.711
PC (14:0/22:6)	0.63 (0.55 - 0.72)	<0.001	0.85 (0.74 - 0.98)	0.029	0.85 (0.73 - 0.98)	0.023
PC (16:0/16:0)	0.99 (0.86 - 1.14)	0.877	0.99 (0.85 - 1.14)	0.838	0.99 (0.85 - 1.14)	0.875
PC (16:0/22:5)	0.92 (0.81 - 1.06)	0.256	0.94 (0.82 - 1.07)	0.352	0.93 (0.82 - 1.07)	0.332
Cer (d18:1/16:0)/Cer (d18:1/24:0) ratio	1.04 (0.91 - 1.19)	0.561	1.09 (0.94 - 1.26)	0.255	1.09 (0.94 - 1.27)	0.237
Cer (d18:1/18:0)/Cer (d18:1/24:0) ratio	1.22 (1.06 - 1.41)	0.006	1.21 (1.03 - 1.42)	0.019	1.21 (1.03 - 1.42)	0.020
Cer (d18:1/24:1)/Cer (d18:1/24:0) ratio	1.15 (1.00 - 1.33)	0.046	1.10 (0.95 - 1.27)	0.202	1.10 (0.95 - 1.28)	0.189
Cer (d18:1/16:0) /PC (16:0/22:5) ratio	1.36 (1.18 - 1.55)	<0.001	1.09 (0.95 - 1.26)	0.204	1.09 (0.95 - 1.25)	0.221
Cer (d18:1/18:0) /PC (14:0/22:6) ratio	1.80 (1.57 - 2.06)	<0.001	1.24 (1.07 - 1.43)	0.004	1.24 (1.07 - 1.44)	0.004
Cer (d18:1/18:0)/Cer (d18:1/16:0) ratio	1.22 (1.06 - 1.41)	0.006	1.16 (0.98 - 1.36)	0.079	1.16 (0.98 - 1.36)	0.084

We used age as the time scale for the models

Model 1 Adjusted for body mass index *BMI*, current smoking, ex smoking, education low/high, prevalent asthma, stratified by sex

Model 2 Adjusted for body mass index *BMI*, ex smoking, education low/high prevalent asthma, stratified by sex and current smoking

Both CERT1 and CERT2 were significantly associated with COPD, including both prevalent and incidence cases. For prevalent COPD, CERT1 showed a significant association in the univariable model (OR = 1.81, 95% CI: 1.41–2.33, *p* < 0.001), but this association did not remain significant after adjustment (adjusted OR = 1.27, 95% CI: 0.96–1.70, *p* = 0.095). In contrast, CERT2 was significantly associated with prevalent COPD in both univariable and adjusted models (adjusted OR 1.57 95% CI: 1.15–2.16). For incident COPD, CERT1 was significantly associated in the univariable analysis (HR = 1.33, 95% CI: 1.16–1.53, *p* < 0.001), and CERT2 also showed a significant association (HR = 1.53, 95% CI: 1.32–1.77, *p* < 0.001). However, neither score remained statistically significant after adjustments in Model 2): CERT1, *p* = 0.067, CERT2, *p* = 0.081). In both the prevalence and incidence analyses of COPD, the Cer (d18:1/18:0)/PC (14:0/22:6) ratio was significantly associated with COPD risk across all models (adjusted OR for prevalence 1.37, 95% CI: 1.01–1.86, Model 2 HR for incidence 1.24, 95% CI: 1.07–1.44). In addition, Cer (d18:1/24:1) was significantly associated with prevalent COPD in both univariable and adjusted models, as well as with incident COPD in the univariable model.

We also tested models with an alternative set of covariates (Supplementary Table 5). In the prevalence analyses, models adjusted for age, sex, and smoking status (current and former) showed that both CERT scores remained statistically significant after adjustment. In the incidence models, adjustments were made for HDL, LDL, BMI, current smoking, frequency of physical activity, and hs-CRP. Additionally, alternative models treating smoking as a stratifying variable rather than a covariate were tested. Both CERT scores and the Cer(d18:1/18:0)/PC (14:0/22:6) ratio retained their statistical significance across these models. Importantly, Cer(d18:1/18:0) itself also remained significantly associated with incident COPD in the fully adjusted stratified model (Model 3: HR = 1.29, 95% CI: 1.06–1.56, *p* = 0.012). In contrast, PC (14:0/22:6) was significantly associated with incident COPD in Models 1 and 2 (HR = 0.69 and 0.75, respectively; both *p* < 0.001) but lost statistical significance in the fully adjusted stratified model (Model 3: HR = 0.89, 95% CI: 0.76–1.04, *p* = 0.150).

### Regression analysis of CERT scores and components by sex

Table 3 presents the results of regression analyses evaluating CERT scores and their components for incident COPD among men (A) and women (B). In unadjusted models, CERT1 was significantly associated with COPD risk in both men (HR = 1.33, 95% CI: 1.13–1.56) and women (HR = 1.58, 95% CI: 1.21–2.06); however, these associations lost statistical significance after adjustments. Similarly, CERT2 showed significant associations in unadjusted models for both sexes, but only in men did the association remain significant after adjustment, suggesting that CERT2 may better predict incident COPD in men than in women.

Among men, the Cer (d18:1/18:0)/Cer (d18:1/24:0) ratio remained significantly associated with incident COPD after adjustment (HR = 1.25, 95% CI: 1.04–1.52). Additionally, the Cer (d18:1/18:0)/PC (14:0/22:6) ratio was significantly associated with COPD incidence in men (HR = 1.25, 95% CI: 1.06–1.49). PC (14:0/22:6) was inversely associated with incident COPD in men, both in univariable models (HR = 0.67, 95% CI: 0.57–0.79) and after adjustment (HR = 0.83, 95% CI: 0.70–0.98). In women, this inverse association was observed only in univariable models.

**Table 3:** Results of study on incidence of COPD in men (A) and women (B)

A. Men (*N* = 3581)				
With COPD *N* = 149 (4.2%), Without COPD : *N *= 3432 (95.8%)				
Variable	Unadjusted HR 95% CI	*P*-value	Adjusted^*^ HR 95% CI	*P*-value
CERT1	1.33 (1.13 - 1.56)	<0.001	1.14 (0.96 - 1.35)	0.138
CERT2	1.54 (1.29 - 1.82)	<0.001	1.20 (1.00 - 1.44)	0.048
Cer (d18:1/16:0)	1.21 (1.03 - 1.43)	0.021	1.00 (0.84 - 1.18)	0.983
Cer (d18:1/18:0)	1.32 (1.11 - 1.56)	0.002	1.12 (0.92 - 1.35)	0.251
Cer (d18:1/24:0)	1.03 (0.87 - 1.22)	0.724	0.91 (0.76 - 1.09)	0.306
Cer (d18:1/24:1)	1.17 (0.99 - 1.39)	0.069	0.99 (0.82 - 1.19)	0.891
PC (14:0/22:6)	0.67 (0.57 - 0.79)	<0.001	0.83 (0.70 - 0.98)	0.031
PC (16:0/16:0)	1.13 (0.96 - 1.33)	0.148	1.05 (0.90 - 1.24)	0.525
PC (16:0/22:5)	0.94 (0.80 - 1.11)	0.461	0.92 (0.79 - 1.08)	0.323
Cer (d18:1/16:0)/Cer (d18:1/24:0)	1.20 (1.02 - 1.41)	0.029	1.12 (0.94 - 1.33)	0.191
Cer (d18:1/18:0)/Cer (d18:1/24:0)	1.37 (1.15 - 1.62)	<0.001	1.25 (1.04 - 1.52)	0.019
Cer (d18:1/24:1)/Cer (d18:1/24:0)	1.18 (1.00 - 1.39)	0.048	1.11 (0.94 - 1.31)	0.234
Cer (d18:1/16:0)/PC (16:0/22:5)	1.27 (1.08 - 1.50)	0.004	1.08 (0.92 - 1.27)	0.349
Cer (d18:1/18:0)/PC (14:0/22:6)	1.68 (1.43 - 1.98)	<0.001	1.25 (1.06 - 1.49)	0.010
Cer (d18:1/18:0)/Cer (d18:1/16:0)	1.22 (1.03 - 1.44)	0.023	1.17 (0.97 - 1.41)	0.111
B. Women (*N *= 4081)				
With COPD* N *= 56 (1.4%), Without COPD : *N* = 4025 (98.6%)				
Variable	Unadjusted HR 95% CI	*P*-value	Adjusted^**^HR 95% CI	*P*-value
CERT1	1.58 (1.21 - 2.06)	<0.001	1.18 (0.90 - 1.56)	0.237
CERT2	1.39 (1.06 - 1.83)	0.017	1.05 (0.79 - 1.39)	0.749
Cer (d18:1/16:0)	1.57 (1.18 - 2.07)	0.002	1.12 (0.84 - 1.50)	0.446
Cer (d18:1/18:0)	1.68 (1.26 - 2.24)	<0.001	1.19 (0.86 - 1.65)	0.297
Cer (d18:1/24:0)	1.41 (1.05 - 1.88)	0.022	1.07 (0.79 - 1.45)	0.647
Cer (d18:1/24:1)	1.69 (1.25 - 2.28)	<0.001	1.17 (0.86 - 1.61)	0.316
PC (14:0/22:6)	0.74 (0.57 - 0.97)	0.031	0.89 (0.67 - 1.17)	0.398
PC (16:0/16:0)	0.82 (0.62 - 1.09)	0.180	0.81 (0.60 - 1.10)	0.177
PC (16:0/22:5)	1.02 (0.78 - 1.34)	0.877	0.98 (0.75 - 1.28)	0.899
Cer (d18:1/16:0)/Cer (d18:1/24:0)	1.08 (0.83 - 1.41)	0.572	1.04 (0.79 - 1.38)	0.776
Cer (d18:1/18:0)/Cer (d18:1/24:0)	1.35 (1.03 - 1.77)	0.031	1.12 (0.83 - 1.52)	0.446
Cer (d18:1/24:1)/Cer (d18:1/24:0)	1.20 (0.91 - 1.57)	0.191	1.11 (0.83 - 1.48)	0.484
Cer (d18:1/16:0)/PC (16:0/22:5)	1.42 (1.09 - 1.84)	0.008	1.10 (0.86 - 1.42)	0.447
Cer (d18:1/18:0)/PC (14:0/22:6)	1.70 (1.31 - 2.19)	<0.001	1.22 (0.92 - 1.61)	0.176
Cer (d18:1/18:0)/Cer (d18:1/16:0)	1.34 (1.02 - 1.77)	0.037	1.12 (0.82 - 1.53)	0.494

* Adjusted for BMI, hs-CRP, education (low/high), ex smoking, and prevalent asthma. Stratified by current smoking

** Adjusted for hs-CRP, education (low/high), and prevalent asthma. Stratified by current smoking

*CERT1* and *CERT2* Cardiovascular Event Risk Test Scores 1 and 2, *HR* Hazard Ratio, *Cer* Ceramide, *PC *Phosphatidylcholine

We also conducted sex-specific regression analyses with an alternative set of covariates (Supplementary Table 6). In men, adjustments included HDL, BMI, hs-CRP, a history of diabetes, and physical activity, with smoking treated as a stratifying variable. Under these conditions, the association between CERT2 and incident COPD lost statistical significance, while other findings remained largely consistent with those from the primary models. In women, models were adjusted for hs-CRP with smoking as a stratifying variable; no significant associations were observed after adjustment, consistent with the results from the primary analyses.

### Kaplan-Meier survival curves for CERT1 and CERT2 to predict COPD incidence in smokers and non-smokers

**Fig. 1:**
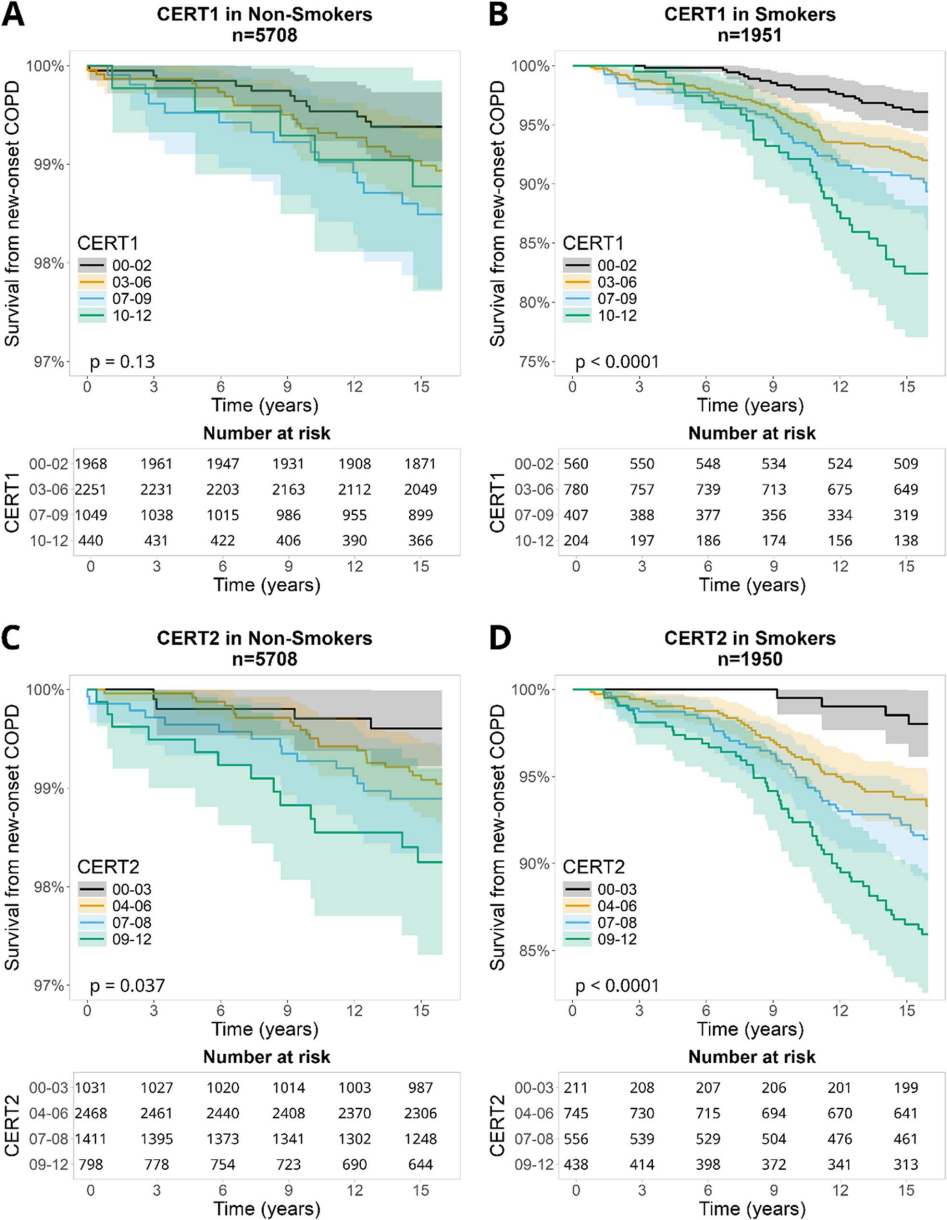
Panels **A** and **C** display Kaplan-Meier curves for Cardiovascular Event Risk Test1 (CERT1) and Cardiovascular Event Risk Test 2 (CERT2) in non-smokers, respectively. Both panels show a gradual increase in the probability of incident Chronic ObstructivePulmonary Disease (COPD), with line colors representing CERT grades: black (00–03),yellow (04–06), blue (07–09), and green (09–12). Panels **B** and **D** show the correspondingcurves for current smokers, where a sharper increase in the probability of incident COPDis observed for higher CERT grades, especially in CERT2. The stronger associationbetween higher CERT grades and incident COPD probability is evident in smokerscompared to the more gradual increase in non-smokers. *P*-values were derived from log-rank tests

Figure 1 displays Kaplan-Meier curves for CERT1 and CERT2 scores, stratified by smoking. In the non-smoker subgroup (Panels A and C, *n* = 5708 for both CERT1 and CERT2), the curves illustrate a gradual increase in the cumulative incidence of COPD over time. Higher CERT grades (shown in blue and green lines) were associated with slightly higher COPD incidence compared with lower grades (black and yellow lines). However, the overall rate of increase in incident COPD among non-smokers remained relatively modest across all CERT categories.

In contrast, among smokers (Panels B and D, *n* = 1951 for CERT1 and *n* = 1950 for CERT2), a pronounced increase in cumulative incidence of COPD was observed, particularly among individuals with higher CERT scores. Smokers in the higher-risk CERT categories experienced a markedly steeper rise in COPD incidence over time compared with those in lower-risk categories, indicating a stronger association between elevated CERT scores and this subgroup. The footnote tables accompanying Fig. 1 further quantify these trends, showing the increasing percentage of incident COPD at various time points and underscoring the amplified impact of smoking between CERT scores and COPD incidence.

## Discussion

Our study demonstrates that CERT scores and Cer components are significantly associated with COPD prevalence and incidence. CERT1 and CERT2 showed significant associations with prevalent and incident COPD, though in most cases the significance was lost after adjusting for confounders, but these findings remain consistent with previous research linking ceramides to lung inflammation, emphysema, and tissue damage [22]. After stratification of the sexes among incident study groups, CERT2 showed stronger associations in men, particularly for incident COPD, suggesting its role in COPD pathophysiology. CERT1 and CERT2 performed better in predicting COPD incidents among smokers than non-smokers. Among the lipids and ratios, Cer (d18:1/24:1) emerged as a key biomarker positively associated with COPD prevalence, while higher PC (14:0/22:6) was inversely associated with COPD incidence, suggesting a potential protective role.

These findings are in agreement with a Korean cohort study that showed elevated Cer levels (d18:1/18:0) and Cer (d18:1/24:1) were associated with reduced FEV1 and prevalent COPD [23], indicating their involvement in the decline of lung function and disease onset. Though in our study, Cer (d18:1/18:0) was only significant in the unadjusted models. In contrast, Cer(d18:1/24:1) was significant in both models in the prevalence study, suggesting that its role in COPD development may be independent of the metabolic changes caused by smoking.

We found that the Cer (d18:1/18:0)/PC (14:0/22:6) ratio was associated with both prevalent and incident COPD, thus serving as a reliable general risk marker for the disease. However, Cer (d18:1/18:0)/Cer (d18:1/24:0) was specifically associated with incident COPD, indicating it may be a prognostic indicator for people at high risk of getting this disease. As a result of these findings, while Cer (d18:1/18:0)/PC (14:0/22:6) may help assess COPD risk generally, Cer (d18:1/18:0)/Cer (d18:1/24:0) might be better suited for predicting COPD incidence.

The findings of our studies are in accordance with those of previous studies suggesting that Cer accumulation in lung tissue contributes to apoptosis and emphysema progression [24]. These results underscore the potential of Cers as early biomarkers of COPD, serving as indicators of both disease presence and progression.

The findings of Mizumura et al. suggest that Cers contribute to cell death and autophagy (self-digestion) in patients with COPD. Following their findings, we evaluated Cer-to-PC ratios, such as Cer (d18:1/18:0)/PC (14:0/22:6), to assess whether they can provide more precise markers for tracking COPD progression and development [25]. These findings suggest the potential utility of Cers in COPD, highlighting the need for further research to validate these biomarkers for early diagnosis and personalized treatment strategies.

### Sex-specific findings and their implications

According to our study, there are sex-based differences in the association between certain Cers and COPD. The association between CERT1, CERT2, and COPD was significant in men in unadjusted models. However, after adjustments, only CERT2, PC (14:0/22:6), Cer (d18:1/18:0)/Cer (d18:1/24:0), and (d18:1/18:0)/PC (14:0/22:6) remained significant, indicating a specific link to COPD risk in men. In the male group, CERT2 also performed a bit better in predicting COPD incidence than CERT1.

In contrast, among women, although initial unadjusted models showed significant associations, none of the CERT scores or components remained significant after adjustment. This discrepancy may partly reflect the smaller number of COPD cases in women (*N* = 56) compared to men (*N* = 149), resulting in lower statistical power for the adjusted models in women. The findings of this study are consistent with those of Ekroos et al., who reported that ceramides and other lipid biomarkers can distinguish cardiovascular and pulmonary disease risks, although these associations vary by sex, age, and other demographic factors. Their review highlights the need to standardize lipidomics to improve disease monitoring, as both our study and theirs illustrate the complex interplay of lipids in COPD and related diseases [26]. Evidence supporting systemic lipid peroxidation and oxidative stress in COPD further strengthens the notion of sex-specific variations in lipid profiles and disease outcomes [27, 28]. These results underscore the importance of developing tailored therapeutic approaches that consider sex differences, as men and women may experience distinct patterns of disease progression.

### Impact of smoking on ceramide metabolism

Kaplan–Meier survival curves indicated that CERT scores predict COPD risk, particularly among smokers. Smoking is well established as the most important risk factor for COPD, and smokers with higher CERT1 and CERT2 scores showed a greater decline in COPD-free survival, highlighting the impact of smoking on ceramide metabolism. Cigarette smoke has been shown to activate the AMPKα1/SMPD3/Cer axis, leading to ceramide accumulation, which contributes to metabolic dysfunction and steatosis [29]. These findings are consistent with our results, suggesting that elevated ceramide levels, especially in smokers, are strongly associated with COPD and underscore the role of ceramides in smoking-induced lung injury and COPD pathogenesis.

Telenga et al. (2014) conducted the only known human study demonstrating both elevated ceramide levels in smokers with COPD and a significant reduction in ceramide expression after two months of smoking cessation, reinforcing the reversible impact of smoking on ceramide metabolism [30]. Although more recent literature, including the review by Kotlyarov [6], supports the mechanistic link between smoking and ceramide dysregulation, no subsequent human studies have replicated the longitudinal findings reported by Telenga et al. Collectively, these studies highlight the contribution of ceramides to smoking-related lung injury and COPD progression in smokers. This evidence suggests that targeting ceramide metabolism may represent a promising therapeutic strategy, although further research is needed to develop clinically applicable treatments.

### Ceramide-phosphatidylcholine interactions

Cer ratios, such as Cer (d18:1/18:0)/PC (14:0/22:6), were significantly associated with both prevalent and incident COPD, with slightly stronger associations observed in incident cases. This highlights the importance of the interaction between Cers and PC in understanding COPD progression. Supporting studies have shown that PC metabolism is tightly regulated in alveolar type II cells to ensure proper surfactant PC synthesis, turnover, and secretion [31]. Although saturated PC levels did not significantly change in some models, the observed ceramide–PC imbalances are consistent with prior findings suggesting that alveolar type II cell dysfunction—particularly impaired surfactant lipid synthesis and secretion—contributes to structural lung changes and COPD pathogenesis [32].

### Linking COPD and cardiovascular disease (CVD)

Our findings indicate that elevated levels of specific Cer species, particularly Cer (d18:1/24:1) for prevalent COPD and the Cer (d18:1/18:0)/Cer (d18:1/24:0) ratio for incident COPD, are significantly associated with the disease. This association is likely driven by the role of Cers in promoting systemic inflammation and oxidative stress, which contribute to COPD pathogenesis. Elevated Cer levels have also been linked to vascular dysfunction, a key feature of CVD, suggesting shared pathogenic mechanisms between COPD and CVD [33]. Research over the past decade has demonstrated that both COPD and CVD share common mechanisms, including chronic low-grade systemic inflammation and endothelial dysfunction. These processes are evident even in early stages of both diseases and tend to worsen during acute exacerbations of COPD, increasing cardiovascular risk [34–36]. These exacerbations increase systemic inflammation, leading to the upregulation of adhesion molecules such as Macrophage-1 Antigen (MAC-1) and inflammatory mediators IL-6 and TNFα. This systemic inflammation contributes to endothelial dysfunction and increases the risk of cardiovascular complications [37–39]. While the lipid profiles of Cers and PCs are similar in both COPD and CVD, certain biomarkers, such as Cer (d18:1/18:0), play distinct roles in the pathogenesis of each condition. Therefore, careful interpretation of these lipid profiles is crucial in clinical settings to differentiate between COPD and CVD, ensuring accurate diagnosis and appropriate treatment strategies. Furthermore, the roles of Cers in systemic inflammation extend beyond COPD and CVD. Recent findings from our work in rheumatoid arthritis (RA) have demonstrated significant associations between CERT scores and inflammation [40], emphasizing the broader relevance of Cers in chronic inflammatory diseases. Similarly, our hypertension studies have identified specific Cer ratios, such as Cer(d18:1/18:0), as significant predictors of the onset of hypertension, reinforcing their role in linking COPD with other non-communicable diseases [41].

Although the diagnosis of COPD in this study adheres to the Finnish national guidelines, as described in the Methods [14], it also aligns with globally accepted standards, such as those outlined in the GOLD 2024 report, which provides evidence-based strategies for the prevention, diagnosis, and management of COPD [42]. It is important to acknowledge the potential variability in the implementation of these guidelines across different healthcare settings. In real-world practice, adherence to clinical guidelines may vary, which could introduce inconsistencies. However, the ascertainment of both prevalent and incident COPD cases in our study was based on in-hospital diagnoses, making these diagnoses highly reliable.

It is probable that among people classified as not having COPD, they may have mild forms of the disease, leading to false negatives. Such false negative cases could weaken or reduce the observed association. However, given that our study included incident cases of COPD over a long follow-up period, the impact of false negatives was minimized to some extent. In addition, the large size of our cohort means that any false negatives among people classified as non-COPD are unlikely to introduce significant bias.

The strength of our study lies in its focus on a cohort of middle-aged individuals, who are at the highest risk of developing COPD. This, however, limits the generalizability of our findings to elderly populations, who may exhibit different Cer profiles or COPD progression. Although we accounted for several confounding factors, there may be residual confounding from unmeasured variables. Additionally, the observational design of the study prevents us from establishing causal relationships between CERT scores and Cer ratios such as Cer (d18:1/18:0) with the COPD outcome. It remains unclear whether systemic inflammation drives Cer release or if elevated Cer levels contribute to inflammation. This underscores the need for longitudinal studies to understand this bidirectional relationship. While Cer (d18:1/18:0) and other Cer/PC ratios were significantly associated with COPD, the precise biological mechanisms underlying these associations remain unclear and warrant further investigations. Although the smoking status was included in our analysis, we could not assess the lifetime cumulative smoking exposure, and smoking habits could have been underreported, potentially influencing the associations observed between Cers and PC with the COPD outcome. In addition, we lacked data on other potential lifestyle and environmental exposures that could contribute to the risk of COPD.

Additionally, individual-level spirometry data such as Forced Expiratory Volume in 1 second (FEV₁) and the FEV₁/FVC ratio were not available in our dataset. As a result, lung function measures could not be included in the baseline characteristics table, which limits the clinical context and precludes a more detailed characterization of the cohort’s pulmonary function at baseline.

Furthermore, we did not have GOLD staging information or other severity-related data to assess or stratify participants by COPD severity. This limitation restricts our ability to adjust analyses for disease stage or to explore whether biomarker associations differ across severity levels. Nevertheless, COPD diagnoses were based on registry-confirmed clinical records in accordance with national and international guidelines that require spirometry, providing confidence in the diagnostic validity.

Furthermore, there is a chance that the regression models will overfit due to the small number of COPD patients, particularly in the prevalence study. The small number of cases may still have an impact on model stability and the generalizability of our findings, even though we reduced the number of covariates and concentrated on clinically justifiable variables to lower this risk.

## Conclusions

This study highlights the potential of CERT1, CERT2, and specific ceramide species — particularly Cer (d18:1/24:1), Cer (d18:1/18:0), and PC (14:0/22:6) — as promising biomarkers for predicting COPD risk and progression. The stronger CERT2 association with COPD in men and the potential impact of Cer (d18:1/18:0) in women indicates sex-specific differences that need further investigation.

The observed associations between the Cer (d18:1/18:0)/PC (14:0/22:6) ratio and COPD incidence highlight the role of these lipid markers in predicting the incidence of COPD in the future and guiding personalized therapeutic approaches. Kaplan-Meier survival further demonstrated the prognostic value of CERT scores in predicting long-term outcomes, particularly in smokers. These findings suggest that Cer and PCs can be effectively measured to assess COPD risk, supporting their relevance in evaluating disease progression. However, further research is needed to better understand how Cers respond to changes in disease activity and various treatments. Integrating these lipid markers into clinical practice may offer opportunities for developing targeted interventions that could slow COPD progression and improve patient outcomes.

## Supplementary Information

Supplementary Material 1.

Supplementary Material 2.

Supplementary Material 3.

Supplementary Material 4.

Supplementary Material 5.

Supplementary Material 6.

Supplementary Material 7.

## Data Availability

The datasets supporting the findings of this study are derived from the FINRISK cohort and are available through the THL Biobank. Access to these datasets is subject to appropriate approval and requires the submission of a data request along with a detailed research plan. Further details on the application process can be found on the THL Biobank data access webpage: https://thl.fi/en/research-and-development/thl-biobank/for-researchers/application-process.

## Abbreviations

ACOS: Asthma-COPD Overlap Syndrome
ApoB: Apolipoprotein B
BMI: Body Mass Index
CERT: Cardiovascular Event Risk Test
CERT1: Cardiovascular Event Risk Test 1
CERT2: Cardiovascular Event Risk Test 2
Cer: Ceramide
Cers: Ceramides
CI: Confidence Interval
COPD: Chronic Obstructive Pulmonary Disease
CVD: Cardiovascular Disease
DALYs: Disability-Adjusted Life Years
FEV1: Forced Expiratory Volume in 1 second
FINRISK: Finnish Institute for Health and Welfare Risk Factor Study
FVC: Forced Vital Capacity
GOLD: Global Initiative for Chronic Obstructive Lung Disease
HDL: High-Density Lipoprotein
HR: Hazard Ratio
hs-CRP: High-Sensitivity C-Reactive Protein
IL-6: Interleukin-6
IQR: Interquartile Range
LDL: Low-Density Lipoprotein
MAC-1: Macrophage-1 Antigen
NSMase: Neutral Sphingomyelinase
OR: Odds Ratio
PC: Phosphatidylcholine
PCs: Phosphatidylcholines
RA: Rheumatoid Arthritis
TNF-α: Tumor Necrosis Factor-alpha
