# Supplementary figures and images for: Exploring the association between ceramide, phosphatidylcholine, and COPD prevalence and incidence: a FINRISK population-based cohort study
Source: BMC Pulm Med. 2025 Oct 15;25:470. doi: 10.1186/s12890-025-03884-7 (PMC12522678; doi:10.1186/s12890-025-03884-7)

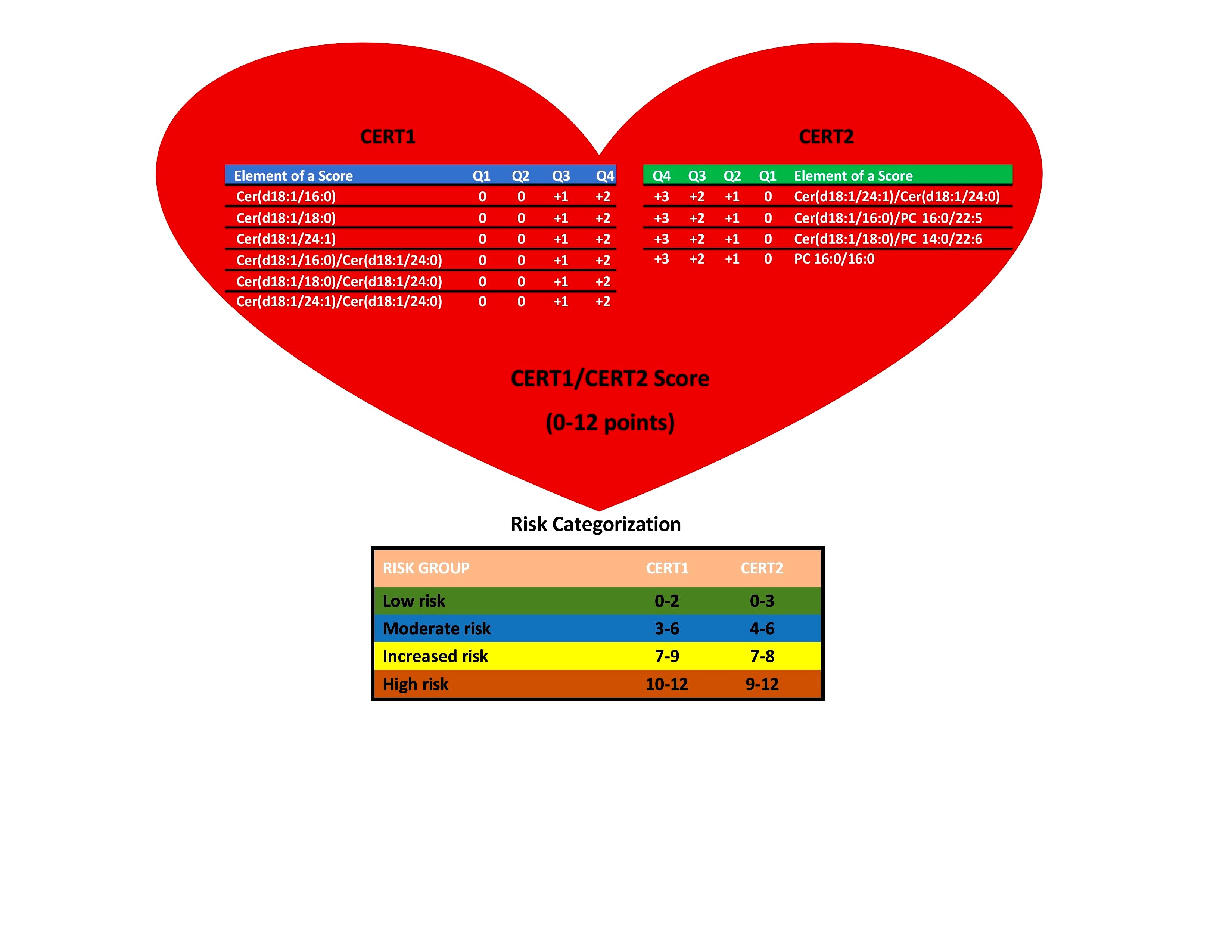

Supplement: Supplementary file 1 — Supplementary Material 1. [file 12890_2025_3884_MOESM1_ESM.jpg]
