# Supplementary material for: Exploring the association between ceramide, phosphatidylcholine, and COPD prevalence and incidence: a FINRISK population-based cohort study
Source: BMC Pulm Med. 2025 Oct 15;25:470. doi: 10.1186/s12890-025-03884-7 (PMC12522678; doi:10.1186/s12890-025-03884-7)
Supplement: Supplementary file 2 — Supplementary Material 2. [file 12890_2025_3884_MOESM2_ESM.docx]

# Supplementary Table 1.

**Percentile cutoff values for CERT1, CERT2, and their component lipid ratios in the FINRISK 2002 cohort (N = 7722). The percentiles divided the data into quartiles of approximately equal size (n = 1930 or 1931, 25.0% each). Minor discrepancies in the number of participants per quartile are due to missing observations, with each lipid having at least one missing value and some having two.**

| **Variable** | **25th percentile** | **50th percentile** | **75th percentile** |
| --- | --- | --- | --- |
| Variables used in CERT1 |  |  |  |
| Cer(d18:1/16:0) | 0.303 | 0.357 | 0.420 |
| Cer(d18:1/16:0)/Cer(d18:1/24:0) | 0.114 | 0.131 | 0.150 |
| Cer(d18:1/18:0) | 0.083 | 0.108 | 0.140 |
| Cer(d18:1/18:0)/Cer(d18:1/24:0) | 0.032 | 0.040 | 0.048 |
| Cer(d18:1/24:1) | 1.392 | 1.670 | 2.011 |
| Cer(d18:1/24:1)/Cer(d18:1/24:0) | 0.544 | 0.615 | 0.695 |
| Variables used in CERT2 |  |  |  |
| Cer(d18:1/16:0)/PC (16:0/22:5) | 0.007 | 0.008 | 0.009 |
| Cer(d18:1/18:0)/PC (14:0/22:6) | 0.076 | 0.109 | 0.160 |
| Cer(d18:1/24:1)/Cer(d18:1/24:0) | 0.544 | 0.615 | 0.695 |
| PC (16:0/16:0) | 17.040 | 19.769 | 23.103 |

**Supplementary Table 1** presents the 25th percentile, median (50th percentile), and 75th percentile cutoff values for the corresponding lipid variables. The total FINRISK 2002 study population analyzed was N = 7,722, resulting in an approximately equal distribution across quartiles (1,930 or 1,931 individuals per group). CERT1 and CERT2 are lipidomics-based cardiovascular risk scores derived from ceramides, phosphatidylcholines, and their ratios: CERT1 incorporates individual ceramides and ceramide-to-ceramide ratios, while CERT2 additionally includes ceramide-to-phosphatidylcholine ratios and selected phosphatidylcholine species, both used for lipid-based risk stratification.
