# Supplementary material for: Exploring the association between ceramide, phosphatidylcholine, and COPD prevalence and incidence: a FINRISK population-based cohort study
Source: BMC Pulm Med. 2025 Oct 15;25:470. doi: 10.1186/s12890-025-03884-7 (PMC12522678; doi:10.1186/s12890-025-03884-7)
Supplement: Supplementary file 3 — Supplementary Material 3. [file 12890_2025_3884_MOESM3_ESM.docx]

**Supplementary Table 2. Associations of lipid biomarkers and clinical variables with COPD: Prevalence (A) and Incidence Analyses(B)**

**Unadjusted and adjusted odds ratios (ORs) with 95% confidence intervals from logistic regression models. Adjusted models include age(log-transformed), hs-CRP, current and ex-smoking, and prevalent asthma.**

| **Variable** | **Univariable OR (95%CI)** | **Univariable P-value** | **Adjusted OR (95%CI)** | **Adjusted P-value** |
| --- | --- | --- | --- | --- |
| CERT1 | 1.81(1.41 - 2.33) | **<0.001** | 1.27(0.96 - 1.70) | 0.095 |
| CERT2 | 2.18(1.67 - 2.87) | **<0.001** | 1.57(1.15 - 2.16) | **0.005** |
| Cer(d18:1/16:0) | 1.59(1.25 - 2.01) | **<0.001** | 1.22(0.92 - 1.62) | 0.164 |
| Cer(d18:1/18:0) | 1.72(1.33 - 2.24) | **<0.001** | 1.14(0.85 - 1.54) | 0.390 |
| Cer(d18:1/24:1) | 1.84 (1.44 - 2.34) | **<0.001** | 1.39 (1.02 - 1.89) | **0.036** |
| Cer(d18:1/24:0) | 1.41 (1.09 - 1.83) | **0.009** | 1.17 (0.87 - 1.57) | 0.304 |
| PC (14:0/22:6) | 0.98(0.76 - 1.26) | 0.849 | 0.79(0.60 - 1.05) | 0.104 |
| PC (16:0/16:0) | 1.52(1.21 - 1.89) | **<0.001** | 1.15(0.86 - 1.51) | 0.343 |
| PC (16:0/22:5) | 1.04(0.81 - 1.34) | 0.765 | 1.00(0.77 - 1.31) | 0.990 |
| Cer(d18:1/16:0)/Cer(d18:1/24:0) ratio | 1.11(0.86 - 1.42) | 0.415 | 1.04(0.79 - 1.37) | 0.779 |
| Cer(d18:1/18:0)/Cer(d18:1/24:0) ratio | 1.42(1.10 - 1.83) | **0.008** | 1.02(0.76 - 1.37) | 0.921 |
| Cer(d18:1/24:1)/Cer(d18:1/24:0) ratio | 1.53(1.18 - 1.98) | **<0.001** | 1.20(0.91 - 1.60) | 0.192 |
| Cer(d18:1/16:0)/PC (14:0/22:6) ratio | 1.52(1.18 - 1.95) | **<0.001** | 1.21(0.91 - 1.62) | 0.183 |
| Cer(d18:1/18:0)/PC (14:0/22:6) ratio | 1.45(1.13 - 1.85) | **0.003** | 1.37(1.01 - 1.86) | **0.041** |
| Cer(d18:1/18:0)/Cer(d18:1/16:0) ratio | 1.39(1.07 - 1.80) | **0.014** | 0.98(0.73 - 1.32) | 0.905 |
| Age (years, log) | 1318.23 (216.08 - 10118.77) | **<0.001** | 1252.66(160.98 - 13030.87) | **<0.001** |
| Sex(female) | 0.51(0.30 - 0.85) | **0.012** | 0.65(0.35 - 1.19) | 0.169 |
| Current smoker | 1.46(0.84 - 2.48) | 0.167 | 7.49(3.52 - 16.53) | **<0.001** |
| Ex-smoker | 2.94(1.75 - 4.89) | **<0.001** | 4.63(2.34 - 9.61) | **<0.001** |
| BMI | 1.09(1.04 - 1.14) | 0.114 | 1.02(0.97 - 1.08) | 0.412 |
| hs-CRP | 1.81(1.44 - 2.29) | **<0.001** | 1.11(0.86 - 1.44) | 0.421 |
| Education(low) | 2.49(1.40 - 4.70) | **0.003** | 0.94(0.50 - 1.87) | 0.860 |
| Prevalent asthma | 31.29(17.93 - 57.43) | **<0.001** | 24.74(13.84 - 46.33) | **<0.001** |

A. Unadjusted and adjusted odds ratios (ORs) with 95% confidence intervals (CIs) from logistic regression models for prevalent COPD.
Adjusted models include log-transformed age, high-sensitivity C-reactive protein (hs-CRP), current smoking, ex-smoking, and prevalent asthma. CERT1 and CERT2 refer to validated ceramide-based risk scores; Cer and PC refer to individual ceramide and phosphatidylcholine species or their ratios.

**Supplementary Table 2 (B). Associations of lipid biomarkers and clinical variables with incident COPD**

| **Variable** | **Univariable HR (95%CI)** | **Univariable P-value** | **Model 1 HR (95%CI)** | **Model 1 P-value** | **Model 2 HR (95%CI)** | **Model 2 P-value** |
| --- | --- | --- | --- | --- | --- | --- |
| CERT1 | 1.33 (1.16 - 1.53) | **<0.001** | 1.15 (0.99 - 1.33) | 0.060 | 1.14 (0.99 - 1.32) | 0.067 |
| CERT2 | 1.53 (1.32 - 1.77) | **<0.001** | 1.14 (0.98 - 1.33) | 0.085 | 1.14 (0.98 - 1.33) | 0.081 |
| Cer (d18:1/16:0) | 1.30 (1.13 - 1.50) | **<0.001** | 1.04 (0.89 - 1.21) | 0.673 | 1.03 (0.88 - 1.21) | 0.740 |
| Cer (d18:1/18:0) | 1.39 (1.20 - 1.61) | **<0.001** | 1.14 (0.97 - 1.34) | 0.115 | 1.13 (0.96 - 1.33) | 0.137 |
| Cer (d18:1/24:0) | 1.22 (1.05 - 1.43) | **0.009** | 1.05 (0.88 - 1.26) | 0.615 | 1.03 (0.88 - 1.21) | 0.711 |
| Cer (d18:1/24:1) | 1.18 (1.03 - 1.35) | **0.016** | 1.03 (0.88 - 1.21) | 0.711 | 1.03 (0.88 - 1.21) | 0.711 |
| PC (14:0/22:6) | 1.28 (1.10 - 1.49) | **0.001** | 1.14 (0.97 - 1.34) | 0.115 | 1.15 (0.98 - 1.35) | 0.087 |
| PC (16:0/16:0) | 0.99 (0.86 - 1.14) | 0.877 | 0.99 (0.86 - 1.14) | 0.838 | 0.99 (0.86 - 1.14) | 0.875 |
| PC (16:0/22:5) | 1.05 (0.91 - 1.22) | 0.514 | 1.01 (0.87 - 1.17) | 0.890 | 1.02 (0.88 - 1.18) | 0.847 |
| Cer (d18:1/16:0)/Cer (d18:1/24:0) | 1.04 (0.92 - 1.19) | 0.561 | 1.03 (0.91 - 1.17) | 0.663 | 1.03 (0.91 - 1.17) | 0.659 |
| Cer (d18:1/18:0)/Cer (d18:1/24:0) | 1.20 (1.04 - 1.39) | **0.015** | 1.06 (0.91 - 1.24) | 0.423 | 1.06 (0.91 - 1.24) | 0.429 |
| Cer (d18:1/24:1)/Cer (d18:1/24:0) | 1.15 (1.00 - 1.32) | **0.045** | 1.09 (0.94 - 1.27) | 0.255 | 1.09 (0.94 - 1.27) | 0.237 |
| Cer (d18:1/16:0) /PC (14:0/22:6) | 1.25 (1.08 - 1.45) | **0.003** | 1.12 (0.96 - 1.32) | 0.152 | 1.12 (0.96 - 1.32) | 0.159 |
| Cer (d18:1/18:0) /PC (14:0/22:6) | 1.80 (1.57 - 2.06) | **<0.001** | 1.29 (1.07 - 1.54) | **0.004** | 1.24 (1.07 - 1.44) | **0.004** |
| Cer (d18:1/18:0)/Cer (d18:1/16:0) | 1.22 (1.06 - 1.41) | **0.006** | 1.16 (0.98 - 1.36) | 0.079 | 1.16 (0.98 - 1.36) | 0.084 |
| Sex (female) | 0.34 (0.25 - 0.46) | **<0.001** |  |  |  |  |
| Current smoker | 16.12 (11.66 -22.28) | **<0.001** | 48.28 (25.00 - 93.23) | **<0.001** |  |  |
| Ex-smoker | 0.79 (0.57 - 1.11) | 0.173 | 1.96 (1.38 - 2.76) | **<0.001** | 6.20(3.03 - 12.70) | **<0.001** |
| BMI | 0.96 (0.93 - 0.99) | **0.022** | 0.97 (0.94 - 1.00) | 0.092 | 0.97 (0.94 - 1.00) | 0.091 |
| hs-CRP | 1.47 (1.28 - 1.69) | **<0.001** | 1.19 (1.12 - 1.49) | **<0.001** | 1.20 (1.01 - 1.50) | **<0.001** |
| Education (low) | 2.42 (1.67 - 3.50) | **<0.001** | 1.95 (1.35 - 2.82) | **<0.001** | 1.91 (1.32 - 2.77) | **<0.001** |
| Prevalent asthma | 2.00 (1.38 - 2.89) | **<0.001** | 2.74 (1.88 - 3.99) | **<0.001** | 2.68 (1.84 - 3.92) | **<0.001** |

B. Hazard ratios (HRs) and 95% confidence intervals (CIs) from Cox regression models for incident COPD.

Age was used as the time scale. CERT1 and CERT2 refer to validated ceramide-based risk scores; Cer and PC refer to individual ceramide and phosphatidylcholine species or their ratios.

Model 1: Adjusted for BMI, hs-CRP, education, current and ex-smoking, and prevalent asthma; stratified by sex.

Model 2: As Model 1, but also stratified by current smoking.
