# Supplementary material for: Exploring the association between ceramide, phosphatidylcholine, and COPD prevalence and incidence: a FINRISK population-based cohort study
Source: BMC Pulm Med. 2025 Oct 15;25:470. doi: 10.1186/s12890-025-03884-7 (PMC12522678; doi:10.1186/s12890-025-03884-7)
Supplement: Supplementary file 4 — Supplementary Material 4. [file 12890_2025_3884_MOESM4_ESM.docx]

**Supplementary Table 3**

**Baseline characteristics for prevalent chronic obstructive pulmonary disease (COPD) in participants aged ≥ 50**

**Total participants: 3,641**

| **Variable** | **People without COPD (N=3,582)** | **People with COPD (N=59)** | **P-value** |
| --- | --- | --- | --- |
| Age (years) | 58.9 (54.7–64.1) | 64.6 (58.8–68.3) | **<0.001** |
| Men | 1776 (49.6 %) | 37 (62.7 %) | 0.062 |
| Higher education (%) | 1031 (28.8%) | 14 (23.7%) | 0.480 |
| Serum Total Cholesterol (mmol/L) | 5.79 (5.11–6.49) | 5.70 (4.91–6.25) | 0.159 |
| Serum HDL cholesterol (mmol/L)- Men | 1.29 (1.09–1.54) | 1.21 (1.04–1.44) | 0.299 |
| Serum HDL cholesterol (mmol/L)- Women | 1.60 (1.36–1.92) | 1.66 (1.21–1.79) | 0.298 |
| Serum LDL cholesterol (mmol/L) | 3.49 (2.92–4.08) | 3.34 (2.60–3.71) | 0.058 |
| Serum triglycerides (mmol/L) | 1.29 (0.95–1.83) | 1.57 (1.14–2.11) | **0.017** |
| Serum ApoB (g/L) | 1.02 (0.89–1.19) | 0.97 (0.88–1.17) | 0.335 |
| BMI (Kg/m²) | 27.5 (24.9–30.4) | 28.4 (24.8–33.2) | 0.237 |
| Systolic blood pressure (SBP) (mmHg) | 81 (75–88) | 80 (72–89) | 0.316 |
| Diastolic blood pressure (DBP) (mmHg) | 141 (127–155) | 146 (129–158) | 0.294 |
| Serum hs-CRP (mg/L) | 1.41 (0.72–2.98) | 2.34 (0.89–6.17) | **0.002** |
| Waist Circumference (cm) - Men | 97.5 (91.0–105.5) | 102.0 (94.5–113.0) | 0.082 |
| Waist Circumference (cm) - Women | 86.5 (79.0–95.0) | 97.5 (80.2–104.8) | **0.036** |
| Current Smoking - Men | 446 (25.1 %) | 12 (32.4 %) | 0.410 |
| Current Smoking - Women | 224 (12.4 %) | 7 (31.8 %) | **0.016** |
| Ex-Smoking - Men | 661 (37.2 %) | 20 (54.1 %) | 0.055 |
| Ex-Smoking - Women | 281 (15.6 %) | 7 (31.8 %) | 0.074 |
| History of diabetes | 318 (8.9 %) | 13 (22.0 %) | **<0.001** |
| History of lipid-lowering drug treatment | 503 (14.0 %) | 12 (20.3 %) | 0.235 |
| History of blood pressure-lowering treatment | 918 (25.6 %) | 26 (44.1 %) | **0.002** |
| History of asthma | 344 (9.6%) | 43 (72.9%) | **<0.001** |
| CERT1 | 5 (3–8) | 6 (4–9) | **0.005** |
| CERT2 | 6 (5–8) | 8 (6–9) | **<0.001** |

BMI: Body Mass Index.

HDL: High-Density Lipoprotein;
LDL: Low-Density Lipoprotein;
hs-CRP: High-Sensitivity C-Reactive Protein;
ApoB: Apolipoprotein B.

CERT1: Cardiovascular Event Risk Test 1

CERT2: Cardiovascular Event Risk Test 2

Note: Values are presented as medians (IQRs) for continuous variables and counts (percentage in group) for categorical variables. The former was compared using Mann-Whitney U tests and the latter using Chi squared tests.
