# Supplementary material for: Exploring the association between ceramide, phosphatidylcholine, and COPD prevalence and incidence: a FINRISK population-based cohort study
Source: BMC Pulm Med. 2025 Oct 15;25:470. doi: 10.1186/s12890-025-03884-7 (PMC12522678; doi:10.1186/s12890-025-03884-7)
Supplement: Supplementary file 5 — Supplementary Material 5. [file 12890_2025_3884_MOESM5_ESM.docx]

**Supplementary Table 4**

**Baseline characteristics for incident chronic obstructive pulmonary disease (COPD) in participants aged ≥ 50 Years (A) and participants aged < 50 Years (B)**

**(A) Total participants: 3,582**

| **Variable** | **People without COPD (N=3,421)** | **People with COPD (N=161)** | **P-value** |
| --- | --- | --- | --- |
| Age (years) | 58.8 (54.7–64.1) | 60.7 (56.1–66.7) | **0.004** |
| Men | 1652 (48.3 %) | 124 (77.0 %) | **<0.001** |
| Higher education (%) | 1003 (29.3%) | 28 (17.4%) | **<0.001** |
| Serum Total Cholesterol (mmol/L) | 5.79 (5.11–6.49) | 5.77 (5.06 - 6.30) | 0.392 |
| Serum HDL cholesterol (mmol/L)- Men | 1.29 (1.09–1.54) | 1.28 (1.11 - 1.51) | 0.939 |
| Serum HDL cholesterol (mmol/L)-Women | 1.60 (1.36–1.92) | 1.54 (1.22 - 1.80) | 0.085 |
| Serum LDL cholesterol (mmol/L) | 3.50 (2.91 - 4.08) | 3.43 (3.01 - 4.27) | 0.657 |
| Serum triglycerides (mmol/L) | 1.29 (0.95 - 1.83) | 1.40 (1.06 - 1.92) | **0.023** |
| Serum ApoB (g/L) | 1.02 (0.89 - 1.19) | 1.04 (0.92 - 1.23) | 0.159 |
| BMI (Kg/m²) | 27.5 (25.0 - 30.4) | 27.1 (23.9 - 29.9) | 0.060 |
| Systolic blood pressure (SBP) (mmHg) | 81 (75 - 88) | 81 (75 - 89) | 0.561 |
| Diastolic blood pressure (DBP) (mmHg) | 141 (127 - 155) | 144 (128 - 158) | 0.247 |
| Serum hs-CRP (mg/L) | 1.38 (0.70 - 2.93) | 2.04 (1.06 - 4.12) | **<0.001** |
| Waist Circumference (cm) - Men | 97.5 (91.0 - 105.4) | 99.0 (89.2 - 107.5) | 0.847 |
| Waist Circumference (cm) - Women | 86.5 (79.0 - 95.0) | 87.5 (78.5 - 99.0) | 0.623 |
| Current Smoking - Men | 366 (22.2%) | 80 (64.5%) | **<0.001** |
| Current Smoking - Women | 192 (10.9%) | 32 (86.5%) | **<0.001** |
| Ex-Smoking - Men | 624 (37.8%) | 37 (29.8%) | 0.096 |
| Ex-Smoking - Women | 277 (15.7%) | 4 (10.8%) | 0.565 |
| History of diabetes | 300 (8.8%) | 18 (11.2%) | 0.363 |
| History of lipid-lowering drug treatment | 475 (13.9%) | 28 (17.4%) | 0.256 |
| History of blood pressure-lowering treatment | 875 (25.6%) | 43 (26.7%) | 0.819 |
| History of asthma | 313 (9.1%) | 31 (19.3%) | **<0.001** |
| CERT1 | 5 (3 - 8) | 6 (4 - 9) | **<0.001** |
| CERT2 | 6 (5 - 8) | 7 (6 - 9) | **<0.001** |

**(B)Total participants: 4,080, participants aged < 50 Years**

| **Variable** | **People without COPD (N=4,036)** | **People with COPD (N=44)** | **P-value** |
| --- | --- | --- | --- |
| Age (years) | 37.9 (31.3–43.8) | 43.3 (37.8–46.8) | **<0.001** |
| Men | 1780 (44.1 %) | 25 (56.8 %) | 0.124 |
| Higher education (%) | 2262 (56.0%) | 7 (15.9%) | **<0.001** |
| Serum Total Cholesterol (mmol/L) | 5.21 (4.65–5.90) | 5.49 (4.80–6.31) | 0.141 |
| Serum HDL cholesterol (mmol/L) - Men | 1.29 (1.09–1.53) | 1.19 (0.93–1.72) | 0.856 |
| Serum HDL cholesterol (mmol/L) - Women | 1.58 (1.35–1.86) | 1.27 (1.09–1.56) | **0.002** |
| Serum LDL cholesterol (mmol/L) | 3.10 (2.57–3.68) | 3.24 (2.90–3.99) | 0.245 |
| Serum triglycerides (mmol/L) | 1.07 (0.79–1.54) | 1.31 (0.87–1.93) | **0.031** |
| Serum ApoB (g/L) | 0.90 (0.77–1.06) | 0.95 (0.84–1.14) | 0.059 |
| BMI (Kg/m²) | 25.2 (22.8–28.2) | 24.3 (21.7–28.6) | 0.514 |
| Systolic blood pressure (SBP) (mmHg) | 76 (69–84) | 78 (70–83) | 0.461 |
| Diastolic blood pressure (DBP) (mmHg) | 125 (116–135) | 124 (114–136) | 0.898 |
| Serum hs-CRP (mg/L) | 0.90 (0.41–2.10) | 1.90 (0.74–3.10) | **0.002** |
| Waist Circumference (cm) - Men | 91.5 (84.5–99.0) | 91.0 (81.5–100.5) | 0.480 |
| Waist Circumference (cm) - Women | 78.5 (72.0–87.0) | 80.0 (74.8–87.2) | 0.442 |
| Current Smoking - Men | 632 (35.5 %) | 23 (92.0 %) | **<0.001** |
| Current Smoking - Women | 612 (27.1 %) | 15 (78.9 %) | **<0.001** |
| Ex-Smoking - Men | 332 (18.7 %) | 2 (8.0 %) | 0.270 |
| Ex-Smoking - Women | 392 (17.4 %) | 1 (5.3 %) | 0.277 |
| History of diabetes | 116 (2.9 %) | 2 (4.5 %) | 0.837 |
| History of lipid-lowering drug treatment | 59 (1.5 %) | 0 (0 %) | 0.863 |
| History of blood pressure-lowering treatment | 175 (4.3 %) | 3 (6.8 %) | 0.667 |
| History of asthma | 272 (6.7%) | 3 (6.8%) | 1.000 |
| CERT1 | 3 (1–6) | 5 (3–8) | **<0.001** |
| CERT2 | 5 (4–7) | 7 (5 - 8) | **<0.001** |

BMI: Body Mass Index;
HDL: High-Density Lipoprotein;
LDL: Low-Density Lipoprotein;
hs-CRP: High-Sensitivity C-Reactive Protein;
ApoB: Apolipoprotein B.

CERT1: Cardiovascular Event Risk Test 1

CERT2: Cardiovascular Event Risk Test 2

Note: Values are presented as medians (IQRs) for continuous variables and counts (percentage in group) for categorical variables. The former was compared using Mann-Whitney U tests and the latter using Chi squared tests.
