# Supplementary material for: Exploring the association between ceramide, phosphatidylcholine, and COPD prevalence and incidence: a FINRISK population-based cohort study
Source: BMC Pulm Med. 2025 Oct 15;25:470. doi: 10.1186/s12890-025-03884-7 (PMC12522678; doi:10.1186/s12890-025-03884-7)
Supplement: Supplementary file 6 — Supplementary Material 6. [file 12890_2025_3884_MOESM6_ESM.docx]

**Supplementary Table 5:** **Logistic and Cox regression results for CERT scores and components in prevalent (A) and incident (B) COPD.**

1. **Study on prevalence of COPD (N=7722)**

| **Variable** | **Unadjusted HR (95%CI)** | **Unadjusted P-value** | **Model 1**  **HR (95%CI)** | **Model 1**  **P-Value** | **Model 2**  **HR (95%CI)** | **Model 2**  **P-Value** |
| --- | --- | --- | --- | --- | --- | --- |
| CERT1 | 1.81 (1.41 - 2.33) | **<0.001** | 1.39 (1.08 - 1.81) | **0.011** | 1.33 (1.03 - 1.73) | **0.033** |
| CERT2 | 2.18 (1.67 - 2.87) | **<0.001** | 1.61 (1.22 - 2.13) | **0.001** | 1.50 (1.13 - 1.99) | **0.005** |
| Cer (d18:1/16:0) | 1.59 (1.25 -2.01) | **<0.001** | 1.30 (1.00 - 1.69) | 0.051 | 1.22 (0.93 - 1.59) | 0.153 |
| Cer (d18:1/18:0) | 1.72 (1.33 - 2.24) | **<0.001** | 1.37 (1.03 - 1.81) | **0.029** | 1.29 (0.96 - 1.72) | 0.089 |
| Cer (d18:1/24:0) | 1.41 (1.09 - 1.83) | **0.009** | 1.20 (0.92 - 1.59) | 0.190 | 1.14 (0.86 - 1.51) | 0.376 |
| Cer (d18:1/24:1) | 1.84 (1.44 - 2.34) | **<0.001** | 1.45 (1.09 - 1.91) | **0.010** | 1.37 (1.03 - 1.83) | **0.032** |
| PC (14:0/22:6) | 0.98 (0.76 - 1.26) | 0.849 | 0.77 (0.59 - 1.02) | 0.061 | 0.84 (0.64 - 1.11) | 0.210 |
| PC (16:0/16:0) | 1.52 (1.21 -1.89) | **<0.001** | 1.18 (0.91 - 1.52) | 0.205 | 1.17 (0.90 - 1.50) | 0.230 |
| PC (16:0/22:5) | 1.04 (0.81 -1.34) | 0.765 | 1.00 (0.78 - 1.29) | 0.990 | 1.01 (0.79 - 1.30) | 0.946 |
| Cer (d18:1/16:0)/  Cer (d18:1/24:0) ratio | 1.11 (0.86 -1.42) | 0.415 | 1.07 (0.82 - 1.37) | 0.617 | 1.06 (0.82 - 1.37) | 0.643 |
| Cer (d18:1/18:0)/  Cer (d18:1/24:0) ratio | 1.42 (1.10 -1.83) | **0.008** | 1.21 (0.93 - 1.59) | 0.160 | 1.18 (0.90 - 1.55) | 0.240 |
| Cer (d18:1/24:1)/  Cer (d18:1/24:0) ratio | 1.53 (1.18 -1.97) | **0.001** | 1.23 (0.95 - 1.60) | 0.114 | 1.22 (0.95 - 1.59) | 0.126 |
| Cer (d18:1/16:0) /  PC (16:0/22:5) ratio | 1.52 (1.18 -1.95) | **0.001** | 1.26 (0.98 - 1.62) | 0.077 | 1.17 (0.91 - 1.52) | 0.222 |
| Cer (d18:1/18:0) /  PC (14:0/22:6) ratio | 1.45 (1.13 -1.85) | **0.003** | 1.51 (1.16 - 1.98) | **0.002** | 1.36 (1.03 - 1.78) | **0.029** |
| Cer (d18:1/18:0)/  Cer (d18:1/16:0) ratio | 1.39 (1.07 -1.80) | **0.014** | 1.19 (0.90 - 1.57) | 0.219 | 1.16 (0.87 - 1.53) | 0.315 |

**Model 1**: Adjusted for age (log) and sex.

**Model 2**: Adjusted for age (log), sex, current smoking, and ex-smoking.

**Count on Table 2**

1. **Study on incidence of COPD (N=7662)**

| **Variable** | **Unadjusted HR (95%CI)** | **Unadjusted P-value** | **Model 1**  **HR (95%CI)** | **Model 1**  **P-Value** | | **Model 2**  **HR (95%CI)** | **Model 2**  **P-Value** | **Model 3**  **HR (95%CI)** | **Model 3 P-Value** |
| --- | --- | --- | --- | --- | --- | --- | --- | --- | --- |
| CERT1 | 1.33 (1.16 - 1.53) | **<0.001** | 1.39 (1.21 - 1.60) | | **<0.001** | 1.39 (1.19 - 1.63) | **<0.001** | 1.22 (1.03 - 1.43) | **0.018** |
| CERT2 | 1.53 (1.32 - 1.77) | **<0.001** | 1.49 (1.29 - 1.73) | | **<0.001** | 1.42 (1.21 - 1.68) | **<0.001** | 1.19 (1.01 - 1.41) | **0.041** |
| Cer (d18:1/16:0) | 1.30 (1.13 - 1.50) | **<0.001** | 1.29 (1.12 - 1.49) | | **<0.001** | 1.35 (1.13 - 1.60) | **0.001** | 1.14 (0.95 - 1.37) | 0.145 |
| Cer (d18:1/18:0) | 1.39 (1.20 - 1.61) | **<0.001** | 1.40 (1.21 - 1.63) | | **<0.001** | 1.55 (1.29 - 1.87) | **<0.001** | 1.29 (1.06 - 1.56) | **0.012** |
| Cer (d18:1/24:0) | 1.22 (1.05 - 1.41) | **0.008** | 1.12 (0.96 - 1.30) | | 0.141 | 1.17 (0.97 - 1.41) | 0.098 | 1.02 (0.84 - 1.24) | 0.816 |
| Cer (d18:1/24:1) | 1.38 (1.19 - 1.60) | **<0.001** | 1.28 (1.10 - 1.49) | | **<0.001** | 1.30 (1.09 - 1.55) | **0.004** | 1.09 (0.91 - 1.32) | 0.340 |
| PC (14:0/22:6) | 0.63 (0.55 - 0.72) | **<0.001** | 0.69 (0.60 - 0.79) | | **<0.001** | 0.75 (0.64 - 0.88) | **<0.001** | 0.89 (0.76 - 1.04) | 0.150 |
| PC (16:0/16:0) | 0.99 (0.86 - 1.14) | 0.877 | 1.04 (0.90 - 1.20) | | 0.562 | 1.07 (0.90 - 1.27) | 0.464 | 1.08 (0.91 - 1.29) | 0.378 |
| PC (16:0/22:5) | 0.92 (0.81 - 1.06) | 0.256 | 0.96 (0.84 - 1.10) | | 0.579 | 1.09 (0.93 - 1.28) | 0.289 | 1.05 (0.90 - 1.23) | 0.547 |
| Cer (d18:1/16:0)/Cer (d18:1/24:0) ratio | 1.04 (0.91 - 1.19) | 0.561 | 1.16 (1.01 - 1.34) | | **0.031** | 1.12 (0.96 - 1.30) | 0.160 | 1.10 (0.94 - 1.28) | 0.244 |
| Cer (d18:1/18:0)/Cer (d18:1/24:0) ratio | 1.22 (1.06 - 1.41) | **0.006** | 1.36 (1.18 - 1.57) | | **<0.001** | 1.37 (1.16 - 1.61) | **<0.001** | 1.24 (1.04 - 1.47) | **0.015** |
| Cer (d18:1/24:1)/Cer (d18:1/24:0) ratio | 1.15 (1.00 - 1.33) | **0.046** | 1.19 (1.03 - 1.36) | | **0.018** | 1.12 (0.97 - 1.31) | 0.132 | 1.07 (0.92 - 1.25) | 0.386 |
| Cer (d18:1/16:0) /PC (16:0/22:5) ratio | 1.36 (1.18 - 1.55) | **<0.001** | 1.31 (1.14 - 1.50) | | **<0.001** | 1.15 (0.98 - 1.34) | 0.079 | 1.05 (0.90 - 1.22) | 0.548 |
| Cer (d18:1/18:0) /PC (14:0/22:6) ratio | 1.80 (1.57 - 2.06) | **<0.001** | 1.69 (1.47 - 1.94) | | **<0.001** | 1.56 (1.34 - 1.81) | **<0.001** | 1.23 (1.05 - 1.44) | **0.010** |
| Cer (d18:1/18:0)/Cer (d18:1/16:0) ratio | 1.22 (1.06 - 1.41) | **0.006** | 1.25 (1.08 - 1.44) | | **0.002** | 1.32 (1.11 - 1.56) | **0.002** | 1.18 (0.99 - 1.41) | 0.065 |

**Note: We used age as the time-scale for the models.**

**Model 1: Stratified by sex.**

**Model 2: Adjusted for sex (stratified), high-density lipoprotein (HDL), low-density lipoprotein (LDL), body mass index (BMI), current smoking, hypertension medication, exercise times per week, and high-sensitivity C-reactive protein (hs-CRP; log-transformed).**

**Model 3: Adjusted for Model 2 covariates, but current smoking is included as a stratifying variable rather than a covariate.**
