# Supplementary material for: Exploring the association between ceramide, phosphatidylcholine, and COPD prevalence and incidence: a FINRISK population-based cohort study
Source: BMC Pulm Med. 2025 Oct 15;25:470. doi: 10.1186/s12890-025-03884-7 (PMC12522678; doi:10.1186/s12890-025-03884-7)
Supplement: Supplementary file 7 — Supplementary Material 7. [file 12890_2025_3884_MOESM7_ESM.docx]

**Supplementary Table 6: Results of study on incidence of COPD in men (A) and women (B)**

**A. Men (N = 3581)**

**With COPD: N = 149 (4.2%), Without COPD: N = 3432 (95.8%)**

| **Variable** | **Unadjusted HR 95% CI** | **P-value** | **Adjusted^*^ HR 95% CI** | **P-value** |
| --- | --- | --- | --- | --- |
| CERT1 | 1.33 (1.13 - 1.56) | **<0.001** | 1.12 (0.93 - 1.34) | 0.221 |
| CERT2 | 1.54 (1.29 - 1.82) | **<0.001** | 1.18 (0.98 - 1.43) | 0.086 |
| Cer (d18:1/16:0) | 1.21 (1.03 - 1.43) | **0.021** | 0.99 (0.83 - 1.19) | 0.930 |
| Cer (d18:1/18:0) | 1.32 (1.11 - 1.56) | **0.002** | 1.10 (0.90 - 1.35) | 0.362 |
| Cer (d18:1/24:0) | 1.03 (0.87 - 1.22) | 0.725 | 0.90 (0.74 - 1.08) | 0.261 |
| Cer (d18:1/24:1) | 1.17 (0.99 - 1.39) | 0.071 | 0.96 (0.79 - 1.16) | 0.640 |
| PC (14:0/22:6) | 0.67 (0.57 - 0.79) | **<0.001** | 0.83 (0.69 - 0.99) | **0.036** |
| PC (16:0/16:0) | 1.13 (0.96 - 1.33) | 0.154 | 1.03 (0.86 - 1.23) | 0.762 |
| PC (16:0/22:5) | 0.94 (0.80 - 1.11) | 0.463 | 0.93 (0.78 - 1.11) | 0.413 |
| Cer (d18:1/16:0)/Cer (d18:1/24:0) | 1.20 (1.02 - 1.41) | **0.029** | 1.14 (0.95 - 1.36) | 0.172 |
| Cer (d18:1/18:0)/Cer (d18:1/24:0) | 1.37 (1.15 - 1.62) | **<0.001** | 1.25 (1.02 - 1.52) | **0.032** |
| Cer (d18:1/24:1)/Cer (d18:1/24:0) | 1.18 (1.00 - 1.39) | **0.048** | 1.08 (0.91 - 1.30) | 0.380 |
| Cer (d18:1/16:0)/PC (16:0/22:5) | 1.27 (1.08 - 1.50) | **0.004** | 1.07 (0.89 - 1.28) | 0.482 |
| Cer (d18:1/18:0)/PC (14:0/22:6) | 1.68 (1.43 - 1.98) | **<0.001** | 1.25 (1.04 - 1.50) | **0.016** |
| Cer (d18:1/18:0)/Cer (d18:1/16:0) | 1.22 (1.03 - 1.44) | **0.023** | 1.15 (0.94 - 1.41) | 0.194 |

1. **Women (N = 4081)**

**With COPD: N = 56 (1.4%), Without COPD: N = 4025 (98.6%)**

| **Variable** | **Unadjusted HR 95% CI** | **P-value** | **Adjusted^**^ HR 95% CI** | **P-value** |
| --- | --- | --- | --- | --- |
| CERT1 | 1.58 (1.21 - 2.06) | **<0.001** | 1.23 (0.94 - 1.63) | 0.141 |
| CERT2 | 1.39 (1.06 - 1.83) | **0.017** | 1.04 (0.79 - 1.38) | 0.762 |
| Cer (d18:1/16:0) | 1.57 (1.18 - 2.07) | **0.002** | 1.16 (0.87 - 1.55) | 0.303 |
| Cer (d18:1/18:0) | 1.68 (1.26 - 2.24) | **<0.001** | 1.26 (0.91 - 1.74) | 0.160 |
| Cer (d18:1/24:0) | 1.41 (1.05 - 1.88) | **0.022** | 1.14 (0.84 - 1.54) | 0.399 |
| Cer (d18:1/24:1) | 1.69 (1.25 - 2.28) | **<0.001** | 1.26 (0.92 - 1.72) | 0.155 |
| PC (14:0/22:6) | 0.74 (0.57 - 0.97) | **0.031** | 0.93 (0.71 - 1.22) | 0.613 |
| PC (16:0/16:0) | 0.82 (0.62 - 1.09) | 0.182 | 0.83 (0.62 - 1.12) | 0.232 |
| PC (16:0/22:5) | 1.02 (0.78 - 1.34) | 0.880 | 1.02 (0.78 - 1.33) | 0.901 |
| Cer (d18:1/16:0)/Cer (d18:1/24:0) | 1.08 (0.83 - 1.41) | 0.573 | 1.02 (0.77 - 1.34) | 0.914 |
| Cer (d18:1/18:0)/Cer (d18:1/24:0) | 1.35 (1.03 - 1.77) | **0.031** | 1.14 (0.84 - 1.54) | 0.410 |
| Cer (d18:1/24:1)/Cer (d18:1/24:0) | 1.20 (0.91 - 1.57) | 0.193 | 1.11 (0.83 - 1.49) | 0.474 |
| Cer (d18:1/16:0)/PC (16:0/22:5) | 1.42 (1.09 - 1.84) | **0.008** | 1.11 (0.86 - 1.43) | 0.445 |
| Cer (d18:1/18:0)/PC (14:0/22:6) | 1.70 (1.31 - 2.19) | **<0.001** | 1.20 (0.91 - 1.58) | 0.203 |
| Cer (d18:1/18:0)/Cer (d18:1/16:0) | 1.34 (1.02 - 1.77) | **0.037** | 1.15 (0.84 - 1.56) | 0.384 |

*After adjustment for HDL, BMI, hs-CRP, diabetes, and exercise times per week, with current smoking as a stratifying variable.

** After adjustment for hs-CRP and current smoking as stratifying variable

**CERT1 and CERT2: Cardiovascular Event Risk Test Scores 1 and 2, HR: Hazard Ratio, Cer: Ceramide, PC: Phosphatidylcholine**
